# Perspectives towards End-of-Life Care in the Emergency Department of Tertiary Public Hospitals—A Qualitative Analysis

**DOI:** 10.3390/medicina59030456

**Published:** 2023-02-24

**Authors:** Ling Tiah, Mui Teng Chua, Win Sen Kuan, Alina Tan, Eileen Tay, Rakhee Yash Pal, Chaoyan Dong

**Affiliations:** 1 Accident & Emergency Department, Changi General Hospital, Singapore Health Services, Singapore 529889, Singapore; 2 Emergency Medicine Department, National University Hospital, National University Health System, Singapore 119074, Singapore; 3 Department of Surgery, Yong Loo Lin School of Medicine, National University of Singapore, Singapore 119077, Singapore; 4 Department of Anesthesia, National University Hospital, National University of Singapore, Singapore 119074, Singapore; 5 Education Office, Sengkang General Hospital, Singapore Health Services, Singapore 554886, Singapore

**Keywords:** emergency department, end-of-life care, qualitative design

## Abstract

*Background and Objectives*: End-of-life care in the emergency department (ED) is gaining importance along with the growth in the ageing population and those with chronic and terminal diseases. To explore key stakeholders’ perspectives and experiences regarding end-of-life care in the ED. *Materials and Methods*: A descriptive qualitative study was conducted from November 2019 to January 2020. Study participants were recruited from the EDs of three tertiary hospitals and community care settings in Singapore through purposive sampling. Data collection included focus group discussions with 36 ED staff, 16 community healthcare professionals, and one-on-one semi-structured interviews with seven family members. *Results*: Three main themes and several subthemes emerged from the data analysis. (1) Reasons for ED visits were attributed to patients’ preferences, families’ decisions, limited services and capabilities in the community, and ease of access. (2) Barriers to providing end-of-life management in the ED included: conflicting priorities of staff, cramped environment, low confidence, ineffective communication, and lack of standardised workflows. (3) Discussion about continuity of end-of-life care beyond the ED uncovered issues related to delayed transfer to inpatient wards, challenging coordination of terminal discharge from the ED, and limited resources for end-of-life care in the community. *Conclusions*: Key stakeholders reported challenges and shared expectations in the provision of end-of-life care in the ED, which could be optimised by multidisciplinary collaborations addressing environmental factors and workflows in the ED. Equipping ED physicians and nurses with the necessary knowledge and skills is important to increase competency and confidence in managing patients attending the ED at the end of their lives.

## 1. Introduction

The fast-paced and often chaotic environment in the emergency department (ED) creates challenges in providing care for patients at the end of their lives. The skills and time needed for holistic end-of-life care compete with the priorities of managing acute emergencies [1,2]. However, the ED frequently becomes the dying patient’s gateway to such care [3] when they have unmanageable symptoms, financial issues, or limited access to community resources [4].

Internationally, although the majority preferred to die at home, up to 80% of patients ended up dying in hospitals [5]. In Singapore, even though more than half of cancer patients expressed a preference for death at home [6], only a quarter of deaths in the general population occurred at private residences, with the majority (60%) dying in hospitals [7]. Singapore’s healthcare infrastructure comprises accessible public hospitals with subsidized financing schemes for citizens through the government [8]. Its EDs are gateways for dying patients who require affordable and round-the-clock medical attention. Patients visiting the ED in Singapore pay a flat fee of Singapore dollars varying from $116 to $132 across institutions [9], which covers the consultation, basic investigations, essential treatment and standard medications. Charges incurred from non-standard services such as imaging scans, procedures and medications are excluded.

End-of-life care in the ED is rapidly gaining importance as the ageing population increases globally [10,11]. However, existing literature provides limited information regarding patients’ and families’ experiences of ED end-of-life management [12], and perspectives and barriers experienced by ED and community healthcare professionals [13,14]. To better comprehend these issues, we conducted an exploratory qualitative study grounded in the constructivist paradigm [15] to explore and examine the perspectives and experiences of (i) next-of-kin of patients requiring end-of-life care in the ED; (ii) ED physicians and nurses in the provision of end-of-life care; and (iii) community palliative care providers regarding end-of-life care in the ED.

## 2. Materials and Methods

Study Design and Setting. This study employed a qualitative, exploratory approach, including focus group discussions and individual interviews between November 2019 and January 2020 in three tertiary public hospitals in Singapore. The theoretical framework guiding the study was social cognitive theory [16], which explains the interplay of individual cognitive, behavioral and social context factors that affect end-of-life decision making and healthcare seeking behaviors. Each of the three participating institutions receives an annual ED census of more than 100,000 attendances [17], and is staffed round-the-clock by board-certified ED physicians and non-specialist doctors. At the time the study was conducted, there were seven public general hospitals in Singapore providing tertiary healthcare services with comprehensive medical facilities, in-house specialists and allied health support. The team of investigators for this study were from three general hospitals and, as such, the study participants were recruited from the three institutions.

Selection of Participants. Purposive sampling was used for recruitment of participants. Physicians and nurses from the EDs who had cared for patients at the end of life were invited by email to participate in the study. In addition, we also approached healthcare professionals from non-ED settings such as hospice care, family medicine practice, community hospitals and nursing homes. These community healthcare providers are integral to the continuum of end-of-life care as patients transit from the community to the ED and vice versa.

For the group of next-of-kin participants, the recruiting criteria included: (i) next-of-kin of patients who had received end-of-life care at any of the three EDs; (ii) these next-of-kin were the designated primary caregivers of the patients; and (iii) they had been present at the ED with the patients for at least four hours. This study was approved by the National Healthcare Group Domain Specific Review Board (DSRB reference number 2018/00838, approved on 15 January 2019) and followed the Standards for Reporting Qualitative Research. For ethical considerations, all the participants’ identifiable information was removed during data collection and analysis to ensure information anonymity and confidentiality.

Data Collection and Analysis. The data collection included focus group discussions (FGDs) with healthcare professionals and one-on-one interviews with the next-of-kin. The reason for holding one-on-one interviews was to respect the next-of-kin’s psychological safety. Each session was facilitated by a pair of trained moderators and conducted in a private room at one of the three participating hospitals. Four of the investigators (L.T., A.T., R.Y.P. and C.D.) were involved as moderators. L.T. and R.Y.P. are emergency medicine physicians involved in palliative care initiatives, including research at their respective EDs in the participating hospitals. They did not facilitate sessions where participants were recruited from their EDs to minimize biases during the interviews. A.T. is a non-specialist physician who had worked in the ED of one of the participating hospitals in the past but was no longer a staff there at the time of the study. C.D. is a medical education researcher who is well-experienced in qualitative research and serves as the assistant director of the education office at a non-participating hospital. Both A.T. and C.D. did not have any working relationship with the participants.

The interview questions were developed through an iterative process, from a literature review [18,19,20,21,22] and research studies previously conducted by the team on end-of-life care in the ED [23,24]. The questions aimed to elicit participants’ perceptions about end-of-life care in the ED, such as positive experiences, challenges, barriers, and suggestions for improvement. Different interview guides were used for (i) healthcare professionals working in the ED; (ii) healthcare professionals practicing in non-ED settings; and (iii) next-of-kin of patients at the end of life in the ED (Appendix A).

Audio-recording of all the interviews was performed using an audio recorder (Samson Zoom H100, Samson Technologies, Hicksville, NY, USA). Each session lasted 60 to 90 min. Nine FGDs and seven one-on-one semi-structured interviews involving a total of 59 participants (52 healthcare professionals and seven family members) were conducted (Table 1).

**Figure 1:**
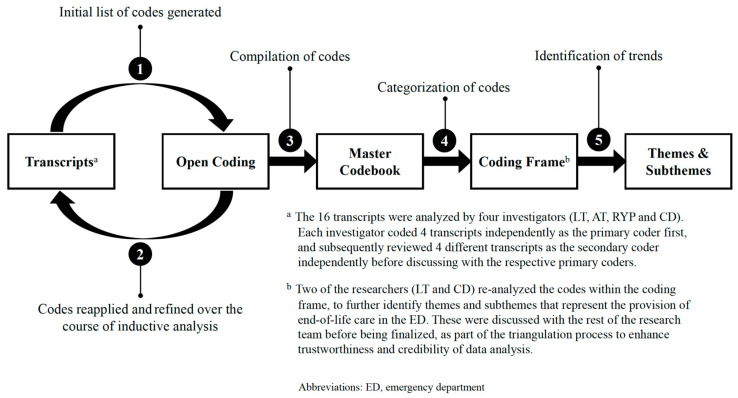
Analysis of data to identify theme.

The data analysis was guided by the constructivist paradigm, and followed the inductive approach [25]. All sessions were recorded, transcribed verbatim, and anonymized before exporting into ATLAS.ti software (ATLAS.ti Scientific Software Development GmbH, Berlin, Germany) for analysis. The 16 transcripts were analyzed by four investigators (L.T., A.T., R.Y.P. and C.D.). Each investigator coded four transcripts independently as the primary coder, and subsequently reviewed four different transcripts as the secondary coder independently before discussing them with the respective primary coders. One pair of researchers (L.T. and C.D.) completed the analysis for the first four transcripts and generated an initial list of codes (open coding). These codes were then applied to the remaining 12 transcripts using the constant comparative method [26], refined by respective pairs of researchers throughout the inductive analysis, and compiled into one master codebook in ATLAS.ti (Figure 1).

**Figure 2:**
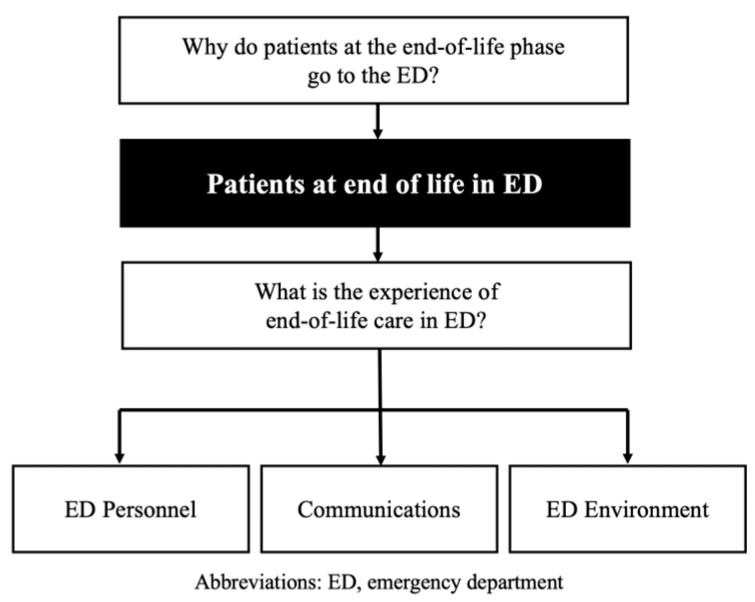
Coding frame.

The codes were next re-examined across the transcripts and broadly categorized based on commonalities into a coding frame. Two of the researchers (L.T. and C.D.) re-analyzed the codes within the coding frame to identify other themes and subthemes representing end-of-life care provision in the ED (Figure 1 and Figure 2). These were discussed with the rest of the research team before being finalized, as part of the triangulation process to enhance the trustworthiness and credibility of data analysis.

## 3. Results

**Figure 3:**
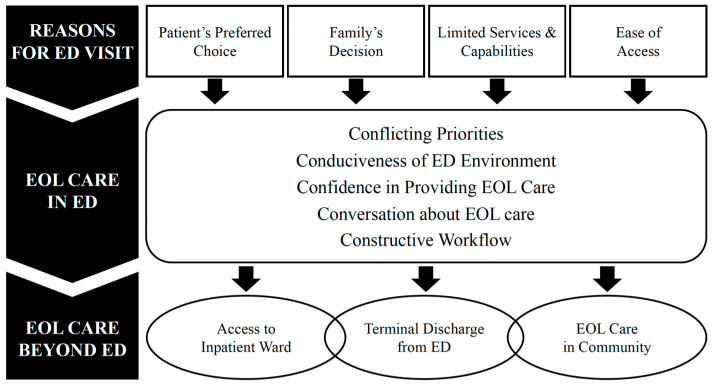
Themes and subthemes.

We identified three main themes with their respective subthemes that contextualized ED visits by patients requiring end-of-life care and their families (Figure 3). Each subtheme with their corresponding supportive quotes are presented as follows. Additional quotes are available as (Appendix B).

### 3.1. Reasons for ED Visit

#### 3.1.1. Patients’ Preferred Choice

One of the common reasons patients in the end-of-life phase were brought to the ED was to be in the hospital when they died. This preference might have been influenced by the assurance of being in a facility with the necessary medication and equipment for symptom management, or prior positive experience of a loved one receiving end-of-life care at the hospital.

“He (the patient) said that no, he is not going to go home …he said ‘I go home, I’m alone… here (there are) people (who) take care of me.’”

(Next-of-kin, Session 4)

#### 3.1.2. Family’s Decision

Family members chose to bring their loved ones who were dying to the ED for various reasons. Participants described families feeling “lost” and “scared”, and sometimes unprepared to accept the impending demise. Prior discussion about the dying phase had not sufficiently prepared them to manage the patient at home during the final days or hours.

“The family is the one who would bring these patients to the ED, because they are very scared… they don’t know what to expect… how the real dying process (is like)...”

(ED physician, Session 1)

Sometimes patients developed acute or new symptoms that the families could not cope with, while some next-of-kin came in hoping for a chance to delay death.

“Family sometimes, because of emotional coping… may change their mind, so they say ‘Let’s give (the patient) another last chance.’”

(Community healthcare professional, Session 7)

#### 3.1.3. Limited Services and Capabilities

Patients were referred for investigations of possible reversible conditions which could not be performed during home care. Similarly, some required procedures for symptomatic relief that primary healthcare facilities could not support. In addition, medications to optimize symptom control were limited in the community. Participants also shared that nursing home staff were not specifically trained to manage end-of-life cases.

“More often than not, it would be a hypotension, desaturation, patients requiring antibiotics that need to be given in the restructured hospital, since the community hospitals have limited antibiotics… Those patients with fentanyl infusions—our nurses are not skilled yet for that.”

(Community healthcare professional, Session 7)

#### 3.1.4. Ease of Access

The ED was described as the gateway to the hospital, serving as all-hours access to comprehensive medical care in the hospital. In addition, admitting the patient provided respite and support for families.

“In the community, I think the services (are) just not enough at the moment. Like home hospice, GPs (general practitioners), they are not available 24/7 and sometimes if you make a referral, it takes a few days...”

(ED physician, Session 3)

### 3.2. End-of-Life Care in the ED

#### 3.2.1. Conflicting Priorities

Participants described challenges faced by ED staff as they attended to patients requiring end-of-life care while managing other critically ill patients. The teams were not able to spend as much time as they would have liked with end-of-life cases. With time and staffing constraints, the needs of these cases were often deemed secondary, especially during peak periods. When patients and their families needed support or updates, they had difficulty finding staff to address their concerns.

“It’s so busy.... It’s so chaotic. I feel the physicians are rushing and they have to attend to emergency cases. You (end-of-life patients) are not so urgent; they will leave you there for a while...”

(Next-of-kin, Session 1)

#### 3.2.2. Conduciveness of ED Environment

Participants felt that a common and open area in the cramped ED was not ideal for end-of-life care. Instead, they agreed that a dedicated private space was essential. In addition, a room big enough to accommodate next-of-kin, instead of a small, shared space separated by curtains or screens, was ideal.

“ED—the space, the environment. It is TOO crowded. I think that is very important, at least give us space…it’s a SHOCK. PATIENT, PATIENT, PATIENT next to each other. And the relatives in between and… the (end-of-life) patient… they are in pain. It’s so painstaking looking at them...”

(Next-of-kin, Session 6)

#### 3.2.3. Confidence in Providing End-of-Life Care

Traditional emergency medicine training focuses on acute life-saving interventions. However, participants observed that many ED staff were inexperienced in the practice of end-of-life care. In addition, there was unfamiliarity with medications prescribed for alleviating end-of-life symptoms, with opioids being a commonly cited class of drug.

“…my patient is gasping, my patient is in severe discomfort, SOB (shortness of breath) and pain, and we are advocating for subcut(aneous) fentanyl or morphine, and they (the physicians) are not comfortable. So, we need to wait for the inpatient (palliative team) to come.”

(ED nurse, Session 6)

#### 3.2.4. Conversations about End-of-Life Care in the ED

Providing episodic care and attending to patients with no prior interaction is integral to emergency medicine. However, in the context of ED end-of-life care, the lack of established rapport made the task particularly challenging.

“It’s very hard to establish an end-of-life (care plan) at the first visit. In a few minutes, it’s very hard to tell the family members, ‘Your mum is going to die and then we are going through the comfort measures.’ Because they will think that we are not doing anything, we are giving up on hope…”

(ED physician, Session 2)

The conversation was difficult when the expected trajectory and prognosis had not been shared with patients and next-of-kin during previous medical encounters. Often, the family was not ready to face the impending demise.

“Sometimes their notes say ‘PALLIATIVE’. But when you go and talk to the patient, talk to the family, they are like ‘Huh? That was never communicated.’ So, it becomes very frustrating for us, and also very scary for the family, and very shocking…”

(ED physician, Session 3)

There was an awareness of the increasing need to have such conversations in the ED with clarity and empathy.

“…it was a young ED physician. I was really impressed with him…While he conveyed it, I think the message (was) clear… I suppose it’s the empathy that comes with it. It’s the human factor—the empathy …”

(Next-of-kin, Session 2)

#### 3.2.5. Constructive Workflow

Protocols and guidelines helped to provide standardized and seamless care. These were useful in identifying and managing patients with end-of-life symptoms, including when to involve the inpatient palliative care team and medical social service department. In addition, advocates and champions for end-of-life care were beneficial in promoting awareness and acting as information resources.

“So, in (Hospital X), there is close collaboration between ED and the palliative care department. During office hours, whenever the patient ends up in the ED who’s actually known to (Hospice X, Hospice Y) or any home care service, they (the ED) will give us a call and we’ll actually go down to see.”

(Community healthcare professional, Session 8)

### 3.3. End-of-Life Care beyond the ED

#### 3.3.1. Access to Inpatient Wards

For patients at the end of life who were planned for admission, timely transfer to an inpatient bed was important for continuity of care. Admission of these patients from the community to inpatient wards could be better streamlined and expedited.

“Recently we worked with (Hospital X) about direct admission… According to the consultant, they will eyeball, see the patient ‘Is he well?’ If he’s well enough, (he) will go direct to the pal(liative) ward in (the hospital) … I personally think it’s a very good way to go.”

(Community healthcare professional, Session 8)

#### 3.3.2. Terminal Discharge from the ED

Terminal discharges required families to be confident and comfortable in managing end-of-life symptoms at home. In addition to emotional preparation and practical training, logistic coordination with community providers was vital. As such, it was challenging to undertake this task from the ED.

“Because the time and amount of resources we spent into organizing ONE terminal discharge, we could have seen maybe five to ten other patients … And because we DON’T do it too frequently, we take EVEN longer...”

(ED physician, Session 3)

#### 3.3.3. End-of-Life Care in the Community

The participants discussed alternatives for end-of-life care, including direct admission to community hospice facilities from homes and nursing homes. To reduce ED attendances, availability and acceptance of these alternatives were necessary.

“Sometimes, we (nursing home) do transfer patients to the inpatient hospice and they pass away there, so we can actually avoid their admission to the ED. Yah. So we… don’t waste resources for a patient who’s able to have direct admission, provided all parties are agreeable...”

(Community healthcare professional, Session 8)

## 4. Discussion

The provision of competent end-of-life care has been recognized to improve the quality of death [27,28]. Its importance cannot be overstated in ensuring minimal suffering to the patient and improve medical, psychological, and relational outcomes [29] to next-of-kin and healthcare professionals [30]. However, despite encountering death daily, the ED has not been associated [31,32] with implementing effective end-of-life care. Our study explored the perspectives of next-of-kin and community healthcare providers, which had not been previously studied. Our study results highlighted the expectations, shortcomings, and potential areas for improvement in the delivery of ED end-of-life care from the lenses of healthcare professionals in the ED and the community, and the next-of-kin.

With the qualitative descriptive research approach, the themes derived from this study are supported by existing theories. According to the Theory of Planned Behavior (TPB) [33,34], an individual’s attitudes, subjective norms, and perceived behavioral control shape underlying behavioral intentions and determine the likelihood of behavior based on evaluating the risks and benefits of the associated behavior outcome. Patients’ attitudes and behavioral intentions and those of their next-of-kin such as inability to cope with new or worsening symptoms, emotional unpreparedness, and belief that hospitals can delay inevitable death, significantly influence patients’ ED attendances.

As illustrated by reciprocal determinism, social influences and the individual’s past experiences, the central concept of Social Cognitive Theory (SCT) [35,36] also explains how one’s behavioral action may be shaped. Participants described positive past experiences of loved ones receiving end-of-life care in the hospital, which subsequently prompted them to seek similar care in the dying phase. Another important factor is the concept of self-efficacy described by SCT [37], like the perceived power and behavioral control outlined in TPB. Many of the next-of-kin expressed deficiencies in skills, knowledge, and confidence in managing end-of-life symptoms such as pain and dyspnea. This sense of helplessness and perceived lack of self-efficacy, behavioral capability, and control result in dying patients being brought to a hospital instead of passing away at home as they may have wished initially. This is further complicated by subjective and social norms (TPB), which suggest that admitting dying family members to the hospital is perceived as helping them. Limitations in community end-of-life care such as the inability to perform procedures and blood tests, lack of round-the-clock services, and inadequate financial subsidy schemes also serve as negative reinforcements (SCT), and obstruct care of the dying at home. A successful home care plan for patients at the end-of-life stage includes educating family members about the expected trajectory and dying phase, training caregivers to manage symptoms, and providing access to community resources.

Provision of end-of-life care in the ED is fraught with challenges [11,38]. Emergency medicine training often adopts a “save-all” mentality. The resulting attitudes and subjective norms cause an unfavorable evaluation of end-of-life care provision in the ED. ED doctors and nurses were impeded by a lack of rapport and confidence in initiating end-of-life conversations, coupled with stress and competing priorities [39,40]. The need to equip the ED team with knowledge and skills to deliver competent end-of-life care was acknowledged. Suggestions included systematic incorporation of end-of-life care training for ED physicians and nurses, to build knowledge, skills, and confidence in end-of-life care [41]. It was also important that the ED team members agreed on the goals of care. Ideally, the ED physician and nurse would engage the patient and family together, facilitating a consistent and unified message and a shared understanding of family dynamics.

Perceived power and control are dampened by unfamiliarity with medical management of end-of-life symptoms, a non-conducive environment, and deficient workflows in aspects such as terminal discharges. A multidisciplinary team consisting of medical social services and palliative care specialists would smoothen the process in these areas [39,42]. In addition, robust support from hospital palliative care teams was critical to improving the coordination of ED terminal discharges, which could be immensely rewarding for all parties involved.

Our findings were similar to previous studies conducted on the perspectives of emergency physicians in providing end-of-life care under different cultural contexts [43,44]. Limited knowledge of palliative care, absence of palliative care specialist input, logistics, time-consuming family discussions, challenges in communication and decision-making due to lack of prior interaction and unpreparedness of families were common barriers identified internationally [45,46,47]. Improvements should be targeted at policies and programs that can affect behavioral change [48,49]. Progress is required in education, financing systems, community training, and workflows to integrate ED and inpatient care [41,42,50,51].

We note limitations in this study. First, due to the inherent nature of a qualitative study, generalizability may be restricted in other healthcare settings. Management of end-of-life patients at the ED is influenced by various factors, such as the healthcare institution’s policy and procedures, the standards of practice in the EDs, the culture and values of the healthcare teams, and the values and preferences of the next-of-kin. All of these bring the challenge of generalizing the findings. Nevertheless, our findings are similar to those of previous studies and add a different perspective from Asia. Second, the participants in our study were voluntarily based, rather than randomly selected to participate. That being said, non-responders may hold different opinions from participants and constitute responder bias. However, given the nature of the study questions, it would not be ethical and possible to make it mandatory for the ED staff, community providers, and next-of-kin to participate in the study. Third, focus group outcomes may be influenced by the group’s dominant member, and group dynamics will influence the quality of the data collected. The moderators also influence how an individual participant, or the group replies to the discussion questions. We minimized these effects by conducting training for the moderators before data collection and having C.D. moderate most of the focus group discussions as her schedule allowed. The moderators also assured participants that all their identifiable information would not be captured during data collection. Fourth, our FDGs included the healthcare professionals working in the ED but did not mix the healthcare professionals with the next-of-kin. We may have missed the interactions between the ED care teams and the next-of-kin. However, to preserve the next-of-kin’ psychological safety, we chose not to mix these participants.

## 5. Conclusions

Key stakeholders reported challenges in and shared expectations about the provision of end-of-life care in the EDs of three public hospitals in Singapore. Patients’ preferences, families’ decisions, limited resources in the community, and ease of access to the ED were common reasons for ED visits. Barriers included conflicting priorities, cramped environment, low confidence, ineffective communication, lack of standardized workflows and time-consuming coordination of terminal discharge from the ED. Multidisciplinary collaborations addressing environmental factors and workflows in the ED are essential to optimize the provision of end-of-life care at the ED. Equipping ED physicians and nurses with the necessary knowledge and skills is equally important in order to augment their competency and confidence in the management of patients attending the ED at the end-of-life phase.

## Figures and Tables

**Table 1:** Participants’ demographic information.

Group	Interview Method	Participants’ Role	Number Recruited (Total)	Age in Years, Median (Interquartile Range)
Physicians from emergency departments	FGD (Session 1) Institution 1	EM physician	6	32.5 (29–40)
		Non-EM physician	0	
	FGD (Session 2) Institution 2	EM physician	6	40.5 (31–50)
		Non-EM physician	0	
	FGD (Session 3) Institution 3	EM physician	4	37.5 (36–40)
		Non-EM physician	1	
Nurses from emergency departments	FGD (Session 4) Institution 1	Nursing managers/clinicians	5	35.0 (32–36)
		Staff nurses	1	
	FGD (Session 5) Institution 2	Nursing managers/clinicians	2	33.0 (31–36)
		Staff nurses	4	
	FGD (Session 6) Institution 3	Nursing managers/clinicians	2	32.0 (30–39)
		Staff nurses	5	
Healthcare professionals from community settings	FGD (Session 7)	Family physician in private practice	2	35.0 (35–42)
		Family physician in polyclinic	1	
		Physician in community hospital	2	
	FGD (Session 8)	Family physician in private practice	1	40.5 (38–45)
		Physician in nursing home	2	
		Physician in hospice care	1	
		Nurse in hospice home care	2	
	FGD (Session 9)	Family physician in private practice	2	46.5 (37–54)
		Physician in nursing home	1	
		Physician in hospice care	1	
		Nurse in hospice home care	1	
Next-of-kin of patients at end of life	One-on-one semi-structured interviews	Children of patients at end of life	5	57.0 (46–58) ^a^
		In-laws of patients at end of life	2	

^a^ Information not available for one next-of-kin. Abbreviations: EM, emergency medicine; FGD, focused group discussion. EM physicians are board-certified emergency medicine specialists; non-EM physicians are doctors working in the emergency department who are either non-trainees or not board-certified specialists (medical officers, locums, resident physicians, or residents-in-training).

## Data Availability

Data is contained within the article and Appendix A and Appendix B.

## Appendix A

End-Of-Life Management Protocol Offered Within Emergency Room (EMPOWER) Study [23].

Interview Guide 1 for Focus Group Discussion—Emergency Department Physicians and Nurses.

Questions:

1. Why do patients come to the Emergency Department at the end-of-life?
2. How do you feel about the end-of-life care given at the Emergency Department?
3. Do you think there are any areas that could be improved upon?
4. Do you have any suggestions on how we can improve end-of-life care in the Emergency Department?
5. Apart from coming to the Emergency Department, do patients have any other options at the end-of-life?

Prompts for Questions 2, 3 and 4:

- Environment
- Staff (doctors, nurses, allied health, others)
- Investigations done
- Treatment given
- Communication/engagement regarding patient’s condition, symptoms, and treatment
- Overall care including spiritual needs and emotional support

Helpful probes:

- Can you talk about that more?
- Help me understand what you mean
- Can you give an example?

If the conversation gets off topic, restate the purpose of the research.

Interview Guide 2 for Focus Group Discussion—Community Healthcare Professionals.

Questions:

1. Why do you need to send patients at end-of-life to the Emergency Department?
2. How do you feel about the end-of-life care given at the Emergency Department?
3. Do you think there are any areas that could be improved upon?
4. Do you have any suggestions on how we can improve end-of-life care in the Emergency Department?
5. Apart from coming to the Emergency Department, do patients have any other options at the end-of-life?

Prompts for Questions 2, 3 and 4:

- Environment
- Staff (doctors, nurses, allied health, others)
- Investigations done
- Treatment given
- Communication/engagement regarding patient’s condition, symptoms, and treatment
- Overall care including spiritual needs and emotional support

Helpful probes:

- Can you talk about that more?
- Help me understand what you mean
- Can you give an example?

If the conversation gets off topic, restate the purpose of the research.

Interview Guide 3 for One-on-One Semi-Structured Interview—Next-of-kin.

Questions:

1. Why did your family member need to come to the Emergency Department on (visit date)?
2. How do you feel about the end-of-life care given at the Emergency Department?
3. Do you think there are any areas that could be improved upon?
4. Do you have any suggestions on how we can improve end-of-life care in the Emergency Department?
5. Apart from coming to the Emergency Department, do patients have any other options at the end-of-life?

Prompts for Questions 2, 3 and 4:

- Environment
- Staff (doctors, nurses, allied health, others)
- Investigations done
- Treatment given
- Communication/engagement regarding patient’s condition, symptoms, and treatment
- Overall care including spiritual needs and emotional support

Helpful probes:

- Can you talk about that more?
- Help me understand what you mean
- Can you give an example?

If the conversation gets off topic, restate the purpose of the research.

## Appendix B

**Table A1:** Additional quotes from participants for identified themes.

Theme	Subtheme	Quotes
Reasons for EOL visit	Patient’s choice: Prefer to die in a hospital	“I feel that when sent to hospital, … there’s somebody there for you—the doctors, the nurses, (there is) medicine, then you feel more secure.”—ED Nurse, Session 5 “I asked Dad, ‘At your last moments, can we have it in the hospital?’… He was very happy with the arrangement (to pass on in hospital) … ‘I can bring you home, but you must be aware that I will not be able to administer the pain relief, medication, oxygen …’ So, I think he might have a bit of a struggle … maybe I want to die at home but I know that I will be in pain and discomfort.”—NOK, Session 2
	Family’s decision: Emotional unpreparedness	“They (the family) have this book (“Spending the last days together”), everything was discussed, but when you talk to them, they feel very lost. Maybe it was explained, but they don’t digest it. So, I believe the reason why they still send patient to the hospital is because they know, they understand, but they don’t know how to do it. They need guidance; they need help.”—ED Nurse, Session 5
	Family’s decision: Inability to cope with new or worse symptoms	“Uncontrolled pain, as in, for a patient who is a palliative or onco(logy) patient, could be like they were very acutely dyspneic, … the family members got anxious, and they didn’t know how to cope with that.”—ED Physician, Session 2 “… people can suddenly turn either septic, or they may suddenly turn breathless, or they get overloaded from the ESRF (end-stage renal failure) … things which they are not prepared for, and because things happen so fast and the goals of care have not even been discussed yet, and the family is understandably very worried, we’ll be forced to send them (to ED).”—Community HCP, Session 7 “Usually old people like [death] to be very peaceful and go at home. So, we actually decided to (keep) her at home. But at that moment, she’s struggling because she’s (mimics gasping for air). She’s struggling. So, do we want to see her struggle in this way to go? No. So that’s why.”—NOK, Session 7
	Family’s decision: Delay death	“Maybe family, at the point of time when the patient suddenly deteriorated, they cannot handle the situation or they are not ready to let go, although they know it’s time to go.”—ED Nurse, Session 4 “I think they (family) think that at least they send to the hospital, the hospital can still assist them, can still treat (the patient). So that even though the patient is dying … by sending the patient to hospital, maybe their life can extend a bit longer.”—ED Nurse, Session 5
	Limited services and capabilities: Unable to perform certain tests and treatment in community	“Sometimes, we also refer (to ED) when there is something acutely reversible that we can’t do at home, (like) blood tests and drips.”—Community HCP, Session 7 “In the community hospital, pain, breathlessness [are common], but we needed to send to ED because we needed some procedure to be done, say, for example, he had a symptomatic pleural effusion that suddenly accumulated, but he had otherwise good function.”—Community HCP, Session 7
	Ease of access: More financial support in acute hospitals	“Technically, if they call the right person [general practitioner], there’s someone who will come down, BUT the cost will be exponentially high, so USUALLY they will end up in A&E (Accident & Emergency) still.”—Community HCP, Session 7 “I have a number of patients who due to insurance... so for the hospice, you can’t use insurance to pay, whereas in hospital, you can.”—Community HCP, Session 8
	Ease of access: Round-the-clock services in ED	“The other reason why they go back to the A&E (Accident & Emergency) is sometimes it’s a cold call and (the patient) is actively dying and unfortunately, we (general practitioner) can’t go down ourselves, so the easiest way is to go back to A&E (Accident & Emergency).”—Community HCP, Session 7 “They cannot cope at home, and primary care cannot provide them with the service at such short notice—because to effect, to link up with a home hospice or anything, it will take time.”—Community HCP, Session 7
	Ease of access: Respite and support for families	“I think I was so tired because I had been running non-stop. And prior to that, I was watching him round the clock. So I was just exhausted. I admittedly was just glad that the doctors were taking care of him for me because we were so tired …”—NOK, Session 2 “We help them to settle everything, from casket to the certificate. So, (there is) less hassle for the family actually.”—ED Nurse, Session 5
EOL care in ED	Conflicting priorities: Juggling between EOL and other critically ill patients	“And I feel like even if I couldn’t do more, I feel like I HAVE to do more. So, for me, I feel that personally it’s a bit difficult, and I think that it’s even worse when there’s no time, like we have to take care of the patient and, you know, that we have to tear ourselves between these things. But unfortunately, that’s just the way it is.”—ED Physician, Session 3 “Because in ED, there are so many cases and there are SO MANY things going on, so there’s only so much you can do in that … (trails off). Yes, you need to care for the patient, but there are so many patients that you need to divide (your time) up and there are only so many people (ED staff) there, so you have to do the best you can in the time that you have.”—ED Physician, Session 2
	Conflicting priorities: Time and manpower constraints	“I believe, on average, when I have had these conversations without specialist support, it takes me—just the discussion alone—about half an hour to forty-five minutes, just the initial initiation of the discussion. But that’s my own experience. So, actually, the time investment is very large.”—ED Physician, Session 1 “But if we are really very busy, we just let them (family members) stay with the patient … Sometimes it may be that you are also not able to provide them the emotional support that they need at that point of time. But sometimes, you can’t blame us, we have other emergencies, we have other patients also… for me, honestly, I don’t prioritize the emotional aspect.”—ED Nurse, Session 6
	Conflicting priorities: Lack of updates	“Of course, I want to know but there is no nurse. Everybody is busy.”—NOK, Session 3 “There’s no nurse that comes by, and before that, there were a lot of people roaming around, but after that, they (the patient and family) are just left there. So, I think the presence of a nurse coming in to check in... I think it makes a difference as well. Of course, having that private space is important, but when it’s already put apart from the whole ED, it makes them feel like ‘Oh, we are abandoned now and there’s no one.’ ”—Community HCP, Session 8
	Conduciveness of ED environment: Overcrowding in ED	“Well, I think for patients who are like her (the dying patient), it may be better that they are actually isolated somewhere else. Yeah, in a room whereby it’s not so hectic (and) to see all the things happening like physicians running around … during the time she was there (in the ED), she was quite stressed because she kept looking around, seeing the physicians doing this and that. And... I think there was a patient who was shouting a lot and she was stressed.”—NOK, Session 5
	Conduciveness of ED environment: Need for privacy and adequate space	“Because if it is actually the last moments, most of our patients will want to be in the presence of their loved ones and a private space for them to mourn, to say what they need to say. (A private space) is actually important instead of the chaotic situation in the ED.”—Community HCP, Session 8 “The only thing about that room is I think it’s a bit small. I mean the room is okay, but we had so many of us. Almost 20 of us including my cousins … So (we) ended up standing along the corridor. So, it was inconvenient for the staff. We also felt that we are blocking (the passageway)—the patients are moving, the physicians are moving, the nurses are moving … it was very congested.” —NOK, Session 5
	Confidence in providing EOL care: Unfamiliar with EOL care and medications	“I find it more difficult, not just clinically difficult to manage, but also (the) family (is) difficult to manage … I’m not … (sighs) I guess I AM equipped to talk to them, and this is my training, but I still … find them a challenge.”—ED Physician, Session 3 “I feel like sometimes the physicians themselves dare not do the, ordering of the medications for the management of the patients, because some think, ‘The patient is too ill. I cannot start fentanyl for the patient because the patient’s blood pressure is already so low. It will further deteriorate the patient.’ ”—ED Nurse, Session 6
	Confidence in providing EOL care: More training necessary	“And the next thing is nursing skill. A lot of them tell me that they are not TRAINED or didn’t undergo a course to insert a subcut(aneous) needle. So, they are not qualified, because they need to get the paper qualification nowadays. So, training and all are also relevant in order to administer palliative treatment in ED.”—Community HCP, Session 8
	Conversation about EOL care in ED: Challenging due to lack of rapport	“In an emergency setting, … the challenge is we are seeing this patient probably for the first time. We look through all the records, we don’t know what the prior discussions have been like and what the patient’s trajectory has been in the last few months. So, it’s a bit challenging for us to take on the role of exploring all these expectations, if they were not previously explored.”—ED Physician, Session 1
	Conversation about EOL care in ED: Family not ready or did not understand prior discussion	“The hardest is (with) these cases, like (when the) patient doesn’t know the diagnosis or … the family doesn’t know and (only) the patient knows it. Yeah, so that’s the hardest.”—ED Nurse, Session 5 “The most difficult are the family who … at the last critical moment, break down and change their mind absolutely. Initially (they) say palliate all the way. Then, when the moment comes and the person is actively dying, all those discussions get thrown out of the window...”—Community HCP, Session 8
	Conversation about EOL care in ED: Importance of empathy and effective communications	“Actually, the physicians were very good. They explained very clearly and they were very tactful. And especially (when) they knew it’s the end-of-life, they explained in a very nice way and gave us a brochure (“Spending the last days together”) … to get us prepared.”—NOK, Session 7
	Conversation about EOL care in ED: Importance of aligning goals of care within ED team	“Actually, you feel lost, because you don’t know what has been conveyed and what has not been conveyed, who accepted it properly, who didn’t accept it properly, and what can I say next? … So you have to go back to your physician, ‘Physician, how? What happened? What are the things that you said?’… Even (for) that, we don’t have (clear) communication between each other.”—ED Nurse, Session 4
	Constructive workflow: Protocols and guidelines for standardized care	“There are also suggestions on the protocol (on) what medications you can give for whatever symptoms that develop during the end-of-life process, and THAT helps us to cognitively offload quite a bit, so I don’t have to think, ‘Oh, what was this drug that I have to give? How much was the dose?’ And everything is written in already and the nurses know how to execute it.”—ED Physician, Session 3 “So, we have a workflow, the EOL pathway, so by referring to that pathway, it’s quite straight(forward)… Even though I’m trained, I do forget because we don’t have these kinds of cases frequently, so it’s good to just refer [to it] ‘Oh, these are the SOP (Standards of Practice) and this is the management.’ ”—ED Nurse, Session 4
	Constructive workflow: Importance of multi-disciplinary collaborations	“Generally, during office hours, we tend to call the pal(liative) team, because they are most familiar with the patients and they do assist us a lot, in terms of, like, with the demands of both ED care, as well as having to care for a pal(liative) patient.”—ED Physician, Session 2 “They (the medical social workers) have come [to the ED] and done the appropriate things, helped us with the (EOL) journey, … they have done a very great job being that person that I needed to be, but I couldn’t be there.”—ED Physician, Session 3
	Constructive workflow: Presence of advocates and champions	“I try to advocate. Like, for example, there’s a patient that I really identify as (being at the) end-of-life, I will advocate to my physicians … There should be nurses who (are) around every shift that can really advocate for them, because we have an end-of-life pathway, but not everyone is aware and not all physicians are aware.”—ED Nurse, Session 6
EOL care beyond ED	Access to inpatient wards: Importance of streamlining access	“When you want to transfer the EOL patient from ED to ward, can the ED doctor decide to send to the EOL room in the ward or not? You see, to me, I think it’s very important, because to send to (any bed in) the General Ward, there are (other) patients in the room and, you know, it defeats the whole purpose of what you are doing in the ED actually. There is no continuation on that.”—Community HCP, Session 8 “The one that I call is the palliative team in the hospital and tell them ‘This case is coming (from nursing home) just for palliation. He’s in the A&E (Accident & Emergency) right now. Can you quickly go and fish him out and just palliate him BEFORE anyone inserts an IV (intravenous) for him?’ But in order to do that, you need to put in that EXTRA effort to communicate with the hospital.”—Community HCP, Session 8
	Terminal discharge from ED: Coordinating logistics is challenging in ED	“It’s JUST a lot of work, you have to call a lot of people.”—ED Physician, Session 3 “I think for terminal discharge, the procedure is really very time-consuming, because it involves (caregiver) education on the medication, sourcing for resources and family education to look after the symptoms.”—ED Nurse, Session 4
	Terminal discharge from ED: Increase in ED terminal discharges	“Some even discharge with morphine, with Paracet Sup (Paracetamol suppository), some of these basic things, and they get a generic list from the A&E (Accident & Emergency) about who to call in the event of demise, so it’s quite good now. And some of the A&E (Accident & Emergency) doctors have training in DipPal (Diploma in Palliative Medicine), so they may even give fentanyl (infusion) pumps.”—Community HCP, Session 7 “So, NOW, increasingly, we are seeing a lot of … discussion on end-of-life care from A&E (Accident & Emergency) … At least for me, I’m seeing a lot more willingness to allow the patient to quickly go back [home] to pass on.”—Community HCP, Session 7
	Terminal discharge from ED: Robust palliative team support required	“Can we refer this kind of patient to the palliative care team? Because they know more about terminal discharges, education, and they recognize the symptoms and can give resource contacts to the patient or the NOK (next-of-kin) … I think it’s good to consider whether we can refer this group of patients or the NOK to the palliative (team), they have more knowledge or more resources. —ED Nurse, Session 4 “For terminal discharges, maybe a specialized group to come in to help us would be better given the ED environment.”—ED Nurse, Session 4
	EOL care in community: Alternatives care arrangements need to be available to avoid ED attendances	“Our community hospital is connected with the restructured hospital. And we wanted to reduce the burden of sending (to ED), reduce the burden of patients. So, what we did was to liaise with the restructured hospital—the (project) group—to see the patients in the community hospital.”—Community HCP, Session 8 “A program done by (the hospital)—giving free phones to elderly who did not have a phone. There is an app on the phone that directly connects patient to the operator. Operators have records and are trained to direct patients according to their needs. For example, if (the) patient is facing a problem, the operator might say okay you have this problem now so I will send someone who is near you to go see you. I guess main point here is to allow patients to have a direct link to services in the community. Now that is lacking. People only know how to call 995 (public ambulance).”—Community HCP, Session 9
	EOL care in community: Family education is important for successful EOL care at home	“I should think that the next leap forward is in family education and home hospice support, in order to deal with that (managing the dying patient at home), rather than providing this care in A&E (Accident & Emergency) … to keep all these people out of hospital.”—ED Physician, Session 1 “Help us (family) to understand the situation, what we can do … let the family know that ‘Anytime (soon) your mom will be going off.’ And what we can do for her.”—NOK, Session 3

Abbreviations: ED, emergency department; EOL, end-of-life; HCP, healthcare professional; NOK, next-of-kin.

## References

[1] Chan G.K. (2004). End-of-life Models and Emergency Department Care. Acad. Emerg. Med..

[2] Clarke R. (2008). Improving end-of-life care in emergency departments. Emerg. Nurse.

[3] Solberg L.M., Hincapie-Echeverri J. (2015). Palliative Care in the Emergency Department. Crit. Care Nurs. Clin. N. Am..

[4] Mierendorf S.M., Gidvani V. (2014). Palliative Care in the Emergency Department. Perm. J..

[5] Alqahtani A.J., Mitchell G. (2019). End-of-Life Care Challenges from Staff Viewpoints in Emergency Departments: Systematic Review. Healthcare.

[6] Lee A., Pang W.S. (1998). Preferred place of death—A local study of cancer patients and their relatives. Singap. Med. J..

[7] (2020). Registry of Births and Deaths, Singapore Demographic Bulletin, July–September 2020.

[8] Lim J. (2017). Sustainable Health Care Financing: The Singapore Experience. Glob. Policy.

[9] Teo J. (2021). Singapore Public Hospitals Raised A&E Fees This Year. The Straits Time. https://www.straitstimes.com/singapore/health/public-hospitals-raised-fees-for-emergency-services-this-year.

[10] Ouchi K., George N., Schuur J.D., Aaronson E.L., Lindvall C., Bernstein E., Sudore R.L., Schonberg M.A., Block S.D., Tulsky J.A. (2019). Goals-of-Care Conversations for Older Adults with Serious Illness in the Emergency Department: Challenges and Opportunities. Ann. Emerg. Med..

[11] Swenson A., Hyde R. (2021). Understanding patients’ end-of-life goals of care in the emergency department. J. Am. Coll. Emerg. Physicians Open.

[12] McCallum K.J., Jackson D., Walthall H., Aveyard H. (2018). Exploring the quality of the dying and death experience in the Emergency Department: An integrative literature review. Int. J. Nurs. Stud..

[13] Sopcheck J., Tappen R.M. (2021). Nursing Home Resident, Family, and Staff Perspectives on Hospital Transfers for End-of-Life Care. Omega J. Death Dying.

[14] Trahan L.M., Spiers J.A., Cummings G.G. (2016). Decisions to Transfer Nursing Home Residents to Emergency Departments: A Scoping Review of Contributing Factors and Staff Perspectives. J. Am. Med. Dir. Assoc..

[15] Allen J.A. (1994). The Constructivist Paradigm. J. Teach. Soc. Work.

[16] Bandura A. (1986). Social Foundations of Thought and Action: A Social Cognitive Theory.

[17] (2020). Attendances at Emergency Medicine Departments.

[18] Selman L., Robinson V., Klass L., Khan S., George R., Shepherd K., Burman R., Koffman J. (2016). Improving confidence and competence of healthcare professionals in end-of-life care: An evaluation of the ‘Transforming End of Life Care’ course at an acute hospital trust: Table 1. BMJ Support. Palliat. Care.

[19] Weng T.C., Yang Y.C., Chen P.J., Kuo W.F., Wang W.L., Ke Y.T., Hsu C.C., Lin K.C., Huang C.C., Lin H.J. (2017). Implementing a novel model for hospice and palliative care in the emergency department: An experience from a tertiary medical center in Taiwan. Medicine.

[20] Di Leo S., Alquati S., Autelitano C., Costantini M., Martucci G., De Vincenzo F., Kuczynska B., Morini A., Trabucco L., Ursicelli R. (2019). Palliative care in the emergency department as seen by providers and users: A qualitative study. Scand. J. Trauma Resusc. Emerg. Med..

[21] Marck C.H., Weil J., Lane H., Weiland T.J., Philip J., Boughey M., Jelinek G.A. (2014). Care of the dying cancer patient in the emergency department: Findings from a National survey of Australian emergency department clinicians. Intern. Med. J..

[22] Smith A.K., Fisher J., Schonberg M.A., Pallin D., Block S.D., Forrow L., Phillips R.S., McCarthy E.P. (2009). Am I Doing the Right Thing? Provider Perspectives on Improving Palliative Care in the Emergency Department. Ann. Emerg. Med..

[23] Pal R.Y., Kuan W.S., Koh Y., Venugopal K., Ibrahim I. (2017). Death among elderly patients in the emergency department: A needs assessment for end-of-life care. Singap. Med J..

[24] Pal R.Y., Kuan W.S., Tiah L., Kumar R., Wong Y.K.Y., Shi L., Zheng C.Q., Lin J., Liang S., Segara U.C. (2020). End-of-life management protocol offered within emergency room (EMPOWER): Study protocol for a multicentre study. BMJ Open.

[25] Thomas D.R. (2006). A general inductive approach for analyzing qualitative evaluation data. Am. J. Eval..

[26] Glaser B.G. (2014). The Constant Comparative Method of Qualitative Analysis. Soc. Probl..

[27] Patrick D.L., Engelberg R.A., Curtis J.R. (2001). Evaluating the Quality of Dying and Death. J. Pain Symptom Manag..

[28] Hales S., Zimmermann C., Rodin G. (2008). The Quality of Dying and Death. Arch. Intern. Med..

[29] Keeley M.P. (2017). Family Communication at the End of Life. Behav. Sci..

[30] Ito Y., Tsubaki M., Fujimoto M., Sakaguchi Y. (2020). Exploring the components of the quality of death in Japanese emergency departments: A qualitative study. Appl. Nurs. Res..

[31] Bailey C., Murphy R., Porock D. (2011). Trajectories of End-of-Life Care in the Emergency Department. Ann. Emerg. Med..

[32] Giles T.M., Hammad K., Breaden K., Drummond C., Bradley S.L., Gerace A., Muir-Cochrane E. (2019). Nurses’ perceptions and experiences of caring for patients who die in the emergency department setting. Int. Emerg. Nurs..

[33] Ajzen I. (2011). The theory of planned behaviour: Reactions and reflections. Psychol. Health.

[34] Icek A., Julius Kuhl J.B. (1985). From Intentions to Actions: A Theory of Planned Behavior.

[35] Bandura A. (1977). Social Learning Theory. Group Organ. Stud..

[36] Riekert K.A., Ockene J.K., Pbert L. (2014). The Handbook of Health Behavior Change.

[37] Harrison A.W., Rainer R.K., Hochwarter W.A., Thompson K.R. (1997). Testing the Self-Efficacy—Performance Linkage of Social—Cognitive Theory. J. Soc. Psychol..

[38] Forero R., McDonnell G., Gallego B., McCarthy S., Mohsin M., Shanley C., Formby F., Hillman K. (2012). A Literature Review on Care at the End-of-Life in the Emergency Department. Emerg. Med. Int..

[39] Grudzen C.R., Richardson L.D., Hopper S.S., Ortiz J.M., Whang C., Morrison R.S. (2011). Does Palliative Care Have a Future in the Emergency Department? Discussions with Attending Emergency Physicians. J. Pain Symptom Manag..

[40] Verhoef M.-J., De Nijs E.J.M., Ootjers C.S., Fiocco M., Fogteloo A.J., Heringhaus C., Marijnen C.A.M., Horeweg N., Van Der Linden Y.M. (2019). End-of-Life Trajectories of Patients With Hematological Malignancies and Patients With Advanced Solid Tumors Visiting the Emergency Department: The Need for a Proactive Integrated Care Approach. Am. J. Hosp. Palliat. Med..

[41] Stone S.C., Mohanty S., Grudzen C.R., Shoenberger J., Asch S., Kubricek K., Lorenz K.A. (2011). Emergency Medicine Physicians’ Perspectives of Providing Palliative Care in an Emergency Department. J. Palliat. Med..

[42] Meier D.E., Beresford L. (2007). Fast Response is Key to Partnering with the Emergency Department. J. Palliat. Med..

[43] Lamba S., Nagurka R., Zielinski A., Scott S.R. (2013). Palliative Care Provision in the Emergency Department: Barriers Reported by Emergency Physicians. J. Palliat. Med..

[44] Aldridge M.D., Hasselaar J., Garralda E., van der Eerden M., Stevenson D., McKendrick K., Centeno C., Meier D.E. (2015). Education, implementation, and policy barriers to greater integration of palliative care: A literature review. Palliat. Med..

[45] Rivera M.R., Torres F.S. (2015). Lack of training and Comfort level with Provision of Palliative Care in Puerto Rican Emergency Departments. Bol. Asoc. Med. Puerto Rico.

[46] Douplat M., Berthiller J., Schott A.M., Potinet V., Le Coz P., Tazarourte K., Jacquin L. (2019). Difficulty of the decision-making process in emergency departments for end-of-life patients. J. Eval. Clin. Pract..

[47] Bailey C.J., Murphy R., Porock D. (2011). Dying cases in emergency places: Caring for the dying in emergency departments. Soc. Sci. Med..

[48] Koh M.Y.H., Lee J.F., Montalban S., Foo C.L., Hum A.Y.M. (2019). ED-PALS: A Comprehensive Palliative Care Service for Oncology Patients in the Emergency Department. Am. J. Hosp. Palliat. Med..

[49] Díaz-Cortés M.D.M., Granero-Molina J., Hernández-Padilla J.M., Rodríguez R.P., Casado M.C., Fernández-Sola C. (2017). Promoting dignified end-of-life care in the emergency department: A qualitative study. Int. Emerg. Nurs..

[50] George N.R., Kryworuchko J., Hunold K.M., Ouchi K., Berman A., Wright R., Grudzen C.R., Kovalerchik O., LeFebvre E.M., Lindor R.A. (2016). Shared Decision Making to Support the Provision of Palliative and End-of-Life Care in the Emergency Department: A Consensus Statement and Research Agenda. Acad. Emerg. Med..

[51] Reuter Q., Marshall A., Zaidi H., Sista P., Powell E.S., McCarthy D.M., Dresden S.M. (2019). Emergency Department-Based Palliative Interventions: A Novel Approach to Palliative Care in the Emergency Department. J. Palliat. Med..

